# 2-Meth­oxy-*N*′-(2-methoxy­benzyl­idene)benzohydrazide

**DOI:** 10.1107/S160053680802521X

**Published:** 2008-08-06

**Authors:** Jiu-Fu Lu, Suo-Tian Min, Xiao-Hui Ji, Zhong-Hai Dang

**Affiliations:** a School of Chemistry and Environmental Science, Shaanxi University of Technology, Hanzhong 723000, People’s Republic of China

## Abstract

The title Schiff base compound, C_16_H_16_N_2_O_3_, was derived from the condensation of 2-methoxy­benzaldehyde with 2-methoxy­benzohydrazide in an ethanol solution. The dihedral angle between the two aromatic rings is 87.5 (3)°. In the crystal structure, the mol­ecules are linked into chains running parallel to the *a* axis by inter­molecular N—H⋯O hydrogen bonds.

## Related literature

For related literature, see: Lu *et al.* (2008*a* ▶,*b* ▶); Nie (2008 ▶); He (2008 ▶); Shi *et al.* (2007 ▶). For bond-length data, see: Allen *et al.* (1987 ▶).
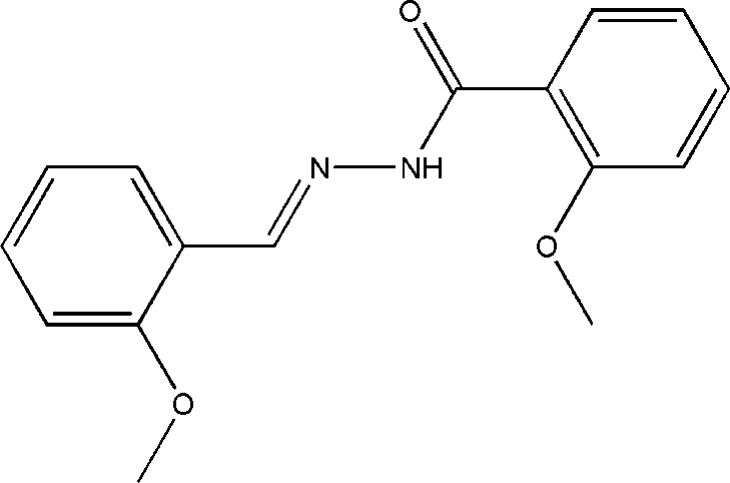


## Experimental

### 

#### Crystal data

C_16_H_16_N_2_O_3_
*M* *_r_* = 284.31
Monoclinic, 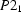

*a* = 4.9998 (13) Å
*b* = 13.475 (4) Å
*c* = 10.824 (3) Å
β = 93.674 (4)°
*V* = 727.7 (4) Å^3^
*Z* = 2
Mo *K*α radiation
μ = 0.09 mm^−1^
*T* = 298 (2) K
0.30 × 0.30 × 0.28 mm

#### Data collection

Bruker APEXII CCD area-detector diffractometer
Absorption correction: multi-scan (*SADABS*; Sheldrick, 2004 ▶) *T*_min_ = 0.973, *T*_max_ = 0.975
6081 measured reflections
1647 independent reflections
1229 reflections with *I* > 2σ(*I*)
*R*_int_ = 0.055

#### Refinement

*R*[*F* ^2^ > 2σ(*F* ^2^)] = 0.050
*wR*(*F* ^2^) = 0.095
*S* = 1.11
1647 reflections
195 parameters
2 restraints
H atoms treated by a mixture of independent and constrained refinement
Δρ_max_ = 0.15 e Å^−3^
Δρ_min_ = −0.14 e Å^−3^

### 

Data collection: *APEX2* (Bruker, 2004 ▶); cell refinement: *SAINT* (Bruker, 2004 ▶); data reduction: *SAINT*; program(s) used to solve structure: *SHELXS97* (Sheldrick, 2008 ▶); program(s) used to refine structure: *SHELXL97* (Sheldrick, 2008 ▶); molecular graphics: *SHELXTL* (Sheldrick, 2008 ▶); software used to prepare material for publication: *SHELXTL*.

## Supplementary Material

Crystal structure: contains datablocks global, I. DOI: 10.1107/S160053680802521X/ci2648sup1.cif

Structure factors: contains datablocks I. DOI: 10.1107/S160053680802521X/ci2648Isup2.hkl

Additional supplementary materials:  crystallographic information; 3D view; checkCIF report

## Figures and Tables

**Table 1:** Hydrogen-bond geometry (Å, °)

*D*—H⋯*A*	*D*—H	H⋯*A*	*D*⋯*A*	*D*—H⋯*A*
N2—H2⋯O2^i^	0.90 (1)	1.99 (1)	2.873 (3)	167 (4)

Symmetry code: (i) 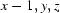.

**Fig. 1.**
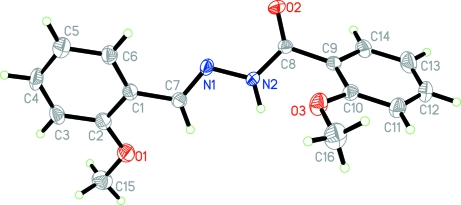
The molecular structure of the title compound, showing 30% probability displacement ellipsoids and the atom-numbering scheme. Dashed lines indicate hydrogen bonds.

**Fig. 2.**
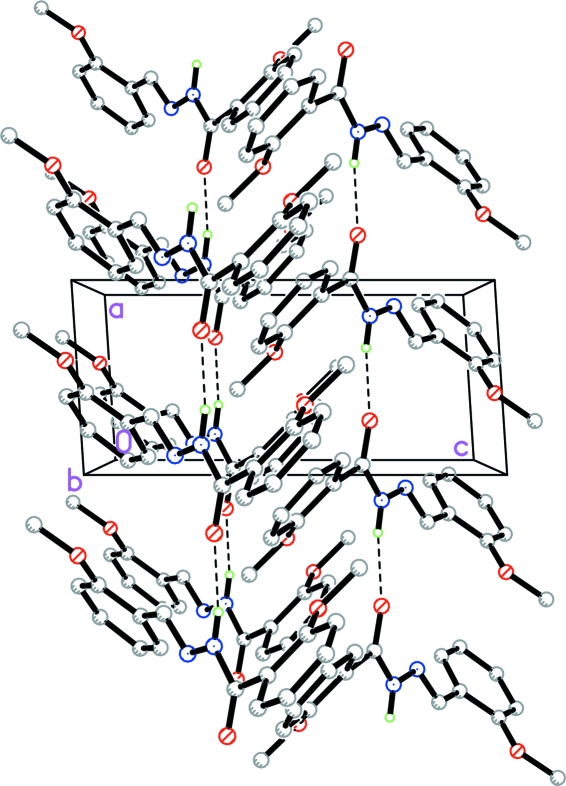
The crystal packing of the title compound, viewed along the b axis. Dashed lines indicate hydrogen bonds.

## supplementary crystallographic information

### Comment

As part of our investigation of the crystal structures of Schiff bases derived from the condensation of aldehydes with benzohydrazides (Lu *et al.*, 2008*a*,b), we report here the crystal structure of the title new Schiff base compound.

In the title molecule (Fig. 1). the bond lengths have normal values (Allen *et al.*,  1987), and are comparable to those observed in related compounds (Nie, 2008; He, 2008; Shi *et al.*,  2007). The dihedral angle between the two aromatic rings is 87.5 (3)°, indicating that they are almost perpendicular to one another.

In the crystal structure, the molecules are linked into chains (Fig. 2) running parallel to the *a* axis by intermolecular N–H···O hydrogen bonds (Table 1).

### Experimental

The title compound was prepared by the Schiff base condensation of 2-methoxybenzaldehyde (0.1 mol) and 2-methoxybenzohydrazide (0.1 mmol) in ethanol (50 ml). The excess ethanol was removed by distillation. The colorless solid obtained was filtered and washed with ethanol. Single crystals suitable for X-ray diffraction were obatined by slow evaporation of an ethanol solution at room temperature.

### Refinement

The imino H atom was located in a difference map and refined with a N–H distance restraint of 0.90 (1) Å. The other H atoms were positioned geometrically (C–H = 0.93-0.96 Å) and refined using a riding model, with U_iso_(H) = 1.2U_eq_(C) and 1.5U_eq_(C_methyl_). In the absence of significant anomalous scattering, Friedel pairs were merged.

### Crystal data

**Table:**

C_16_H_16_N_2_O_3_	*F*_000_ = 300
*M**_r_* = 284.31	*D*_x_ = 1.297 Mg m^−^^3^
Monoclinic, *P*2_1_	Mo *K*α radiation λ = 0.71073 Å
Hall symbol: P 2yb	Cell parameters from 744 reflections
*a* = 4.9998 (13) Å	θ = 2.5–24.0º
*b* = 13.475 (4) Å	µ = 0.09 mm^−^^1^
*c* = 10.824 (3) Å	*T* = 298 (2) K
β = 93.674 (4)º	Block, colourless
*V* = 727.7 (4) Å^3^	0.30 × 0.30 × 0.28 mm
*Z* = 2	

### Data collection

**Table:**

Bruker APEXII CCD area-detector diffractometer	1647 independent reflections
Radiation source: fine-focus sealed tube	1229 reflections with *I* > 2σ(*I*)
Monochromator: graphite	*R*_int_ = 0.055
*T* = 298(2) K	θ_max_ = 27.0º
ω scans	θ_min_ = 1.9º
Absorption correction: multi-scan(SADABS; Sheldrick, 2004)	*h* = −6→6
*T*_min_ = 0.973, *T*_max_ = 0.975	*k* = −17→16
6081 measured reflections	*l* = −13→13

### Refinement

**Table:**

Refinement on *F*^2^	Secondary atom site location: difference Fourier map
Least-squares matrix: full	Hydrogen site location: inferred from neighbouring sites
*R*[*F*^2^ > 2σ(*F*^2^)] = 0.050	H atoms treated by a mixture of independent and constrained refinement
*wR*(*F*^2^) = 0.095	*w* = 1/[σ^2^(*F*_o_^2^) + (0.0297*P*)^2^] where *P* = (*F*_o_^2^ + 2*F*_c_^2^)/3
*S* = 1.11	(Δ/σ)_max_ = 0.001
1647 reflections	Δρ_max_ = 0.16 e Å^−^^3^
195 parameters	Δρ_min_ = −0.14 e Å^−^^3^
2 restraints	Extinction correction: none
Primary atom site location: structure-invariant direct methods	

### Special details

**Table:**

Geometry. All esds (except the esd in the dihedral angle between two l.s. planes) are estimated using the full covariance matrix. The cell esds are taken into account individually in the estimation of esds in distances, angles and torsion angles; correlations between esds in cell parameters are only used when they are defined by crystal symmetry. An approximate (isotropic) treatment of cell esds is used for estimating esds involving l.s. planes.
Refinement. Refinement of F^2^ against ALL reflections. The weighted R-factor wR and goodness of fit S are based on F^2^, conventional R-factors R are based on F, with F set to zero for negative F^2^. The threshold expression of F^2^ > 2sigma(F^2^) is used only for calculating R-factors(gt) etc. and is not relevant to the choice of reflections for refinement. R-factors based on F^2^ are statistically about twice as large as those based on F, and R- factors based on ALL data will be even larger.

### Fractional atomic coordinates and isotropic or equivalent isotropic displacement parameters (Å^2^)

**Table:**

	*x*	*y*	*z*	*U*_iso_*/*U*_eq_	
O1	0.4130 (5)	0.5063 (2)	1.0258 (2)	0.0579 (8)	
O2	1.2673 (4)	0.71624 (19)	0.6930 (2)	0.0436 (6)	
O3	0.6361 (5)	0.7245 (2)	0.4683 (2)	0.0513 (6)	
N1	0.8931 (5)	0.5916 (2)	0.7771 (2)	0.0357 (7)	
N2	0.8339 (5)	0.67811 (19)	0.7116 (3)	0.0354 (7)	
C1	0.7337 (7)	0.4561 (2)	0.8904 (3)	0.0371 (9)	
C2	0.5766 (7)	0.4321 (3)	0.9892 (3)	0.0426 (9)	
C3	0.6008 (7)	0.3398 (3)	1.0439 (3)	0.0501 (10)	
H3	0.4953	0.3233	1.1086	0.060*	
C4	0.7794 (8)	0.2725 (3)	1.0032 (4)	0.0549 (11)	
H4	0.7928	0.2102	1.0400	0.066*	
C5	0.9407 (8)	0.2954 (3)	0.9081 (4)	0.0537 (10)	
H5	1.0645	0.2498	0.8818	0.064*	
C6	0.9142 (7)	0.3876 (3)	0.8529 (3)	0.0455 (9)	
H6	1.0216	0.4036	0.7887	0.055*	
C7	0.6996 (7)	0.5520 (2)	0.8295 (3)	0.0377 (8)	
H7	0.5353	0.5844	0.8291	0.045*	
C8	1.0305 (6)	0.7328 (2)	0.6674 (3)	0.0296 (7)	
C9	0.9390 (6)	0.8198 (2)	0.5913 (3)	0.0343 (8)	
C10	0.7391 (6)	0.8162 (3)	0.4959 (3)	0.0358 (8)	
C11	0.6635 (7)	0.9012 (3)	0.4321 (3)	0.0518 (10)	
H11	0.5257	0.8990	0.3703	0.062*	
C12	0.7913 (8)	0.9890 (3)	0.4597 (4)	0.0606 (12)	
H12	0.7384	1.0462	0.4168	0.073*	
C13	0.9946 (8)	0.9936 (3)	0.5493 (4)	0.0617 (12)	
H13	1.0835	1.0533	0.5659	0.074*	
C14	1.0677 (7)	0.9096 (3)	0.6151 (4)	0.0478 (10)	
H14	1.2059	0.9131	0.6767	0.057*	
C15	0.2563 (8)	0.4878 (4)	1.1267 (4)	0.0653 (12)	
H15A	0.1495	0.4295	1.1105	0.098*	
H15B	0.1410	0.5435	1.1385	0.098*	
H15C	0.3714	0.4778	1.2001	0.098*	
C16	0.4372 (8)	0.7167 (4)	0.3692 (3)	0.0712 (12)	
H16A	0.2821	0.7542	0.3885	0.107*	
H16B	0.3884	0.6483	0.3573	0.107*	
H16C	0.5066	0.7422	0.2949	0.107*	
H2	0.662 (3)	0.696 (3)	0.696 (3)	0.080*	

### Atomic displacement parameters (Å^2^)

**Table:**

	*U*^11^	*U*^22^	*U*^33^	*U*^12^	*U*^13^	*U*^23^
O1	0.0577 (16)	0.0581 (19)	0.0601 (19)	0.0064 (15)	0.0196 (15)	0.0132 (15)
O2	0.0265 (12)	0.0451 (14)	0.0586 (14)	0.0050 (12)	−0.0024 (10)	0.0062 (13)
O3	0.0604 (15)	0.0471 (15)	0.0439 (13)	−0.0052 (14)	−0.0149 (11)	0.0053 (14)
N1	0.0391 (16)	0.0318 (15)	0.0356 (15)	0.0028 (13)	−0.0030 (13)	0.0079 (13)
N2	0.0271 (14)	0.0357 (16)	0.0428 (16)	0.0036 (13)	−0.0018 (13)	0.0124 (13)
C1	0.037 (2)	0.038 (2)	0.0354 (19)	−0.0036 (16)	−0.0070 (16)	0.0053 (16)
C2	0.041 (2)	0.046 (2)	0.040 (2)	−0.0060 (18)	−0.0050 (18)	0.0062 (18)
C3	0.054 (2)	0.051 (3)	0.045 (2)	−0.011 (2)	−0.0020 (18)	0.0176 (19)
C4	0.069 (3)	0.035 (2)	0.058 (3)	0.000 (2)	−0.016 (2)	0.018 (2)
C5	0.061 (3)	0.043 (2)	0.056 (3)	0.0023 (19)	−0.001 (2)	0.001 (2)
C6	0.049 (2)	0.042 (2)	0.045 (2)	−0.0021 (19)	0.0002 (18)	0.0085 (18)
C7	0.0336 (18)	0.043 (2)	0.0357 (19)	0.0002 (16)	−0.0008 (16)	0.0078 (16)
C8	0.0279 (17)	0.0279 (18)	0.0332 (16)	−0.0013 (15)	0.0026 (13)	−0.0029 (15)
C9	0.0317 (17)	0.0346 (19)	0.0377 (18)	0.0046 (15)	0.0098 (14)	0.0017 (16)
C10	0.0368 (19)	0.0353 (19)	0.0357 (19)	0.0037 (16)	0.0051 (15)	0.0015 (17)
C11	0.055 (2)	0.056 (3)	0.044 (2)	0.008 (2)	0.001 (2)	0.013 (2)
C12	0.065 (3)	0.046 (3)	0.071 (3)	0.007 (2)	0.009 (2)	0.027 (2)
C13	0.072 (3)	0.039 (2)	0.076 (3)	−0.010 (2)	0.014 (3)	0.008 (2)
C14	0.048 (2)	0.037 (2)	0.058 (2)	−0.0087 (19)	0.0060 (19)	0.003 (2)
C15	0.060 (3)	0.083 (3)	0.054 (3)	−0.002 (3)	0.012 (2)	0.009 (2)
C16	0.077 (3)	0.078 (3)	0.055 (2)	−0.014 (3)	−0.023 (2)	0.009 (3)

### Geometric parameters (Å, °)

**Table:**

O1—C2	1.367 (4)	C6—H6	0.93
O1—C15	1.407 (4)	C7—H7	0.93
O2—C8	1.220 (3)	C8—C9	1.488 (4)
O3—C10	1.364 (4)	C9—C14	1.387 (5)
O3—C16	1.419 (4)	C9—C10	1.391 (4)
N1—C7	1.270 (4)	C10—C11	1.378 (5)
N1—N2	1.387 (3)	C11—C12	1.368 (5)
N2—C8	1.341 (4)	C11—H11	0.93
N2—H2	0.901 (10)	C12—C13	1.360 (5)
C1—C6	1.370 (5)	C12—H12	0.93
C1—C2	1.405 (5)	C13—C14	1.375 (5)
C1—C7	1.456 (4)	C13—H13	0.93
C2—C3	1.380 (5)	C14—H14	0.93
C3—C4	1.365 (5)	C15—H15A	0.96
C3—H3	0.93	C15—H15B	0.96
C4—C5	1.383 (5)	C15—H15C	0.96
C4—H4	0.93	C16—H16A	0.96
C5—C6	1.381 (5)	C16—H16B	0.96
C5—H5	0.93	C16—H16C	0.96
			
C2—O1—C15	118.0 (3)	C14—C9—C10	118.1 (3)
C10—O3—C16	118.0 (3)	C14—C9—C8	117.5 (3)
C7—N1—N2	115.9 (3)	C10—C9—C8	124.3 (3)
C8—N2—N1	120.5 (2)	O3—C10—C11	123.8 (3)
C8—N2—H2	120 (3)	O3—C10—C9	115.9 (3)
N1—N2—H2	120 (3)	C11—C10—C9	120.3 (3)
C6—C1—C2	118.8 (3)	C12—C11—C10	120.0 (4)
C6—C1—C7	121.6 (3)	C12—C11—H11	120.0
C2—C1—C7	119.6 (3)	C10—C11—H11	120.0
O1—C2—C3	125.0 (3)	C13—C12—C11	120.8 (4)
O1—C2—C1	115.3 (3)	C13—C12—H12	119.6
C3—C2—C1	119.7 (4)	C11—C12—H12	119.6
C4—C3—C2	120.1 (4)	C12—C13—C14	119.6 (4)
C4—C3—H3	120.0	C12—C13—H13	120.2
C2—C3—H3	119.9	C14—C13—H13	120.2
C3—C4—C5	121.1 (3)	C13—C14—C9	121.2 (4)
C3—C4—H4	119.5	C13—C14—H14	119.4
C5—C4—H4	119.5	C9—C14—H14	119.4
C6—C5—C4	118.6 (4)	O1—C15—H15A	109.5
C6—C5—H5	120.7	O1—C15—H15B	109.5
C4—C5—H5	120.7	H15A—C15—H15B	109.5
C1—C6—C5	121.6 (4)	O1—C15—H15C	109.5
C1—C6—H6	119.2	H15A—C15—H15C	109.5
C5—C6—H6	119.2	H15B—C15—H15C	109.5
N1—C7—C1	120.3 (3)	O3—C16—H16A	109.5
N1—C7—H7	119.9	O3—C16—H16B	109.5
C1—C7—H7	119.9	H16A—C16—H16B	109.5
O2—C8—N2	122.8 (3)	O3—C16—H16C	109.5
O2—C8—C9	122.0 (3)	H16A—C16—H16C	109.5
N2—C8—C9	115.1 (3)	H16B—C16—H16C	109.5

### Hydrogen-bond geometry (Å, °)

**Table:**

*D*—H···*A*	*D*—H	H···*A*	*D*···*A*	*D*—H···*A*
N2—H2···O2^i^	0.90 (1)	1.99 (1)	2.873 (3)	167 (4)

Symmetry codes: (i) *x*−1, *y*, *z*.
